# Delirium in intensive care unit patients under noninvasive ventilation: a multinational survey

**DOI:** 10.5935/0103-507X.20150061

**Published:** 2015

**Authors:** Lilian Maria Sobreira Tanaka, Jorge Ibrain Figueira Salluh, Felipe Dal-Pizzol, Bruna Brandão Barreto, Ricardo Zantieff, Eduardo Tobar, Antonio Esquinas, Lucas de Castro Quarantini, Dimitri Gusmao-Flores

**Affiliations:** 1 Hospital Copa D'Or - Rio de Janeiro (RJ), Brazil.; 2 Instituto D'Or de Pesquisa e Ensino - Rio de Janeiro (RJ), Brazil.; 3 Laboratório de Fisiopatologia Experimental and Instituto Nacional de Ciência e Tecnologia Translacional em Medicina, Postgraduate Program in Health Sciences, Unidade Acadêmica de Ciências da Saúde, Universidade do Extremo Sul Catarinense - Criciúma (SC), Brazil.; 4 Postgraduate Program in Health Sciences, Hospital Universitário Prof. Edgar Santos, Faculdade de Medicina da Bahia, Universidade Federal da Bahia - Salvador (BA), Brazil.; 5 Intensive Care Unit, Hospital Universitário Prof. Edgar Santos, Faculdade de Medicina da Bahia, Universidade Federal da Bahia - Salvador (BA), Brazil.; 6 Critical Patient Unit, Departament of Medicine, Hospital Clínico Universidad de Chile - Independencia, Chile.; 7 Department of Intensive Care and Noninvasive Ventilatory Unit, Hospital Morales Meseguer - Murcia, Spain.; 8 Psychiatry Service, Department of Neurosciences and Mental Health, Hospital Universitário Prof. Edgar Santos, Universidade Federal da Bahia - Salvador (BA), Brazil.

**Keywords:** Delirium, Noninvasive ventilation, Diagnostic techniques, neurological, Critical care, Attitude of health personnel, Questionnaires

## Abstract

**Objective:**

To conduct a multinational survey of intensive care unit professionals to determine the practices on delirium assessment and management, in addition to their perceptions and attitudes toward the evaluation and impact of delirium in patients requiring noninvasive ventilation.

**Methods:**

An electronic questionnaire was created to evaluate the profiles of the respondents and their related intensive care units, the systematic delirium assessment and management and the respondents' perceptions and attitudes regarding delirium in patients requiring noninvasive ventilation. The questionnaire was distributed to the cooperative network for research of the *Associação de Medicina Intensiva Brasileira* (AMIB-Net) mailing list and to researchers in different centers in Latin America and Europe.

**Results:**

Four hundred thirty-six questionnaires were available for analysis; the majority of the questionnaires were from Brazil (61.9%), followed by Turkey (8.7%) and Italy (4.8%). Approximately 61% of the respondents reported no delirium assessment in the intensive care unit, and 31% evaluated delirium in patients under noninvasive ventilation. The Confusion Assessment Method for the intensive care unit was the most reported validated diagnostic tool (66.9%). Concerning the indication of noninvasive ventilation in patients already presenting with delirium, 16.3% of respondents never allow the use of noninvasive ventilation in this clinical context.

**Conclusion:**

This survey provides data that strongly reemphasizes poor efforts toward delirium assessment and management in the intensive care unit setting, especially regarding patients requiring noninvasive ventilation.

## Abstract (pt)

**Objetivos:**

Conduzir um inquérito multinacional com profissionais de unidades de terapia intensiva para determinar as práticas relacionadas à avaliação e ao manejo do *delirium*, bem como as percepções e as atitudes relacionadas à avaliação e ao impacto do *delirium* em pacientes submetidos à ventilação não invasiva.

**Métodos:**

Foi elaborado um questionário eletrônico para avaliar o perfil dos respondedores e das unidades de terapia intensiva a eles relacionadas; a realização de avaliação sistemática e a forma de manejo do *delirium*; e as percepções e condutas dos profissionais com relação à presença de *delirium* em pacientes submetidos à ventilação não invasiva. O questionário foi distribuído por meio da mala direta de correio eletrônico da rede de cooperação em pesquisa clínica da Associação de Medicina Intensiva Brasileira (AMIB-Net) e para pesquisadores em diferentes centros da América Latina e Europa.

**Resultados:**

Foram analisados 436 questionários que, em sua maioria, eram provenientes do Brasil (61,9%), seguidos por Turquia (8,7%) e Itália (4,8%). Aproximadamente 61% dos respondedores relataram não proceder à avaliação de *delirium* na unidade de terapia intensiva, enquanto 31% a realizavam em pacientes submetidos à ventilação não invasiva. *Confusion Assessment Method for the Intensive Care Unit* foi a ferramenta diagnóstica validada mais frequentemente citada (66,9%). Com relação à indicação de ventilação não invasiva para pacientes em *delirium*, 16,3% dos respondedores nunca permitiam o uso de ventilação não invasiva neste contexto clínico.

**Conclusão:**

Este inquérito fornece dados que enfatizam a escassez de esforços direcionados à avaliação e ao manejo do *delirium* no ambiente da terapia intensiva, em especial nos pacientes submetidos à ventilação não invasiva.

## INTRODUCTION

Delirium constitutes one of the most frequent complications in hospitalized patients. Its prevalence in the intensive care unit (ICU) varies with the population studied and may be up to 80% in individuals under mechanical ventilation (MV).^(1-3)^ Despite acknowledging that this condition is associated with adverse outcomes, such as long-term cognitive impairment, higher reintubation rates, longer hospital length of stay and mortality,^(3-7)^ delirium is still underdiagnosed; assessment by a validated diagnostic tool, as demonstrated by previous surveys, remains suboptimal.^(1,8)^

Data regarding delirium in patients under noninvasive ventilation (NIV) that include the prognostic impact of delirium in NIV failure are scarce.^(9)^ While successful NIV improves oxygenation and respiratory mechanics and can decrease ICU-acquired complication,^(10)^ NIV failure, in contrast, is associated with increased ICU mortality.^(11,12)^ The development of agitation and the deterioration of mental status, such as in delirious patients, decreases the ability to cooperate and tolerate NIV, potentially increasing the risks for NIV failure and subsequent intubation.^(13)^

We conducted a multinational survey of ICU professionals to determine the practices of delirium assessment and management and their perceptions and attitudes toward the evaluation and impact of delirium in patients requiring NIV.

## METHODS

### Survey development and administration

An Internet survey that evaluated ICU professionals mainly comprising doctors, nurses and physiotherapists was carried out from July to November 2013.

The questionnaire was initially created in Portuguese in May 2013 and identified relevant points on how to manage delirious patients. Specialists in delirium in critically ill patients discussed all questions.

Pilot testing was performed to assure the validity and reliability of the questionnaire. In this phase, intensivists with experience in clinical research were asked to answer all questions in the Internet survey format. Questions that were considered not relevant or difficult to understand were reformulated or deleted. Time taken to answer each question was recorded, and questions that required more than 1 minute to be answered were reassessed. After these adjustments, non-medical professionals with no experience in clinical research assessed the questionnaire to evaluate the question comprehension. One of the researchers was always present at this evaluation. The technique "thinking aloud" (in which the respondent is asked to verbalize thought while responding to a question) was used to ensure an adequate understanding of the question.^(14)^ In the second phase, the questionnaire (Appendix 1S in the electronic supplementary materials) was translated into Spanish and English, following the recommendation of the International Society of Pharmacoeconomic and Outcomes Research (ISPOR) for translation and cultural validation of the questionnaire.^(15)^

These steps resulted in a 3-part questionnaire that evaluated the profiles of respondents and related ICUs (8 questions), the systematic delirium assessment and management (4 questions) and the respondents' perceptions and attitudes regarding delirium in patients requiring NIV (9 questions).

The questionnaires were distributed to the cooperative network for research of the *Associação de Medicina Intensiva Brasileira* (AMIB-Net) mailing list and to researchers in different centers in Latin America and Europe.

The survey did not contain data that could identify the respondents. The institutional review board of the *Universidade Federal da Bahia* (the main institution responsible for the study) approved the study and waived the need for informed consent. All study steps were conducted in compliance with the Declaration of Helsinki.

### Data and statistical analysis

The survey results were exported to a Microsoft Excel^TM^ template and were analyzed using IBM^®^ SPSS^®^ Statistics software package, Version 21.0 for Macintosh (Armonk, NY: IBM Corp). Variables were reported as numbers (percentage). Because the number of respondents varied for each question, the proportions displayed in the results section and the tables are not constant. Fisher's exact test was used for the comparison of the variables. A 2-sided p-value of less than 0.05 was considered significant.

## RESULTS

### Demographics

A total of 436 questionnaires were available for analysis; the majority of the surveys were from Brazil (61.9%), followed by Turkey (8.7%), Italy (4.8%), Chile (3.7%) and Portugal (3.4%). Other participating countries (n = 33) contributed 76 questionnaires (a list of all participating countries is available as Appendix 2S, in the electronic supplementary materials). Demographic characteristics from the survey respondents are depicted in table 1. Respondents mainly comprised physicians (63.8%). Physiotherapists comprised 24.1% of the analyzed professionals, while nurses comprised 10.1%. Irrespective of their education, most of the respondents (above 70%) were board-certified in Intensive Care Medicine, and 55.1% of the respondents had 1 to 10 years of ICU experience.

**Table 1:** Demographic characteristics from survey respondents and intensive care units

Characteristics	N (%)
Board-certification in intensive care medicine	334 (76.6)
Physician	218/278 (78.4)
Physiotherapist	77/105 (73.3)
Nurse	31/44 (70.5)
Years of ICU practice	
1 - 5	121 (27.8)
5 - 10	119 (27.3)
> 10	196 (45)
Main practice setting	
Private institution	159 (36.4)
Public institution	121 (27.8)
Academic medical center	156 (35.8)
ICU type*	
Medical	122 (28)
Surgical	82 (18.8)
Neurological	52 (11.9)
Mixed	265 (60.8)
ICU size	
< 10 beds	182 (41.7)
11 - 20 beds	136 (31.2)
> 20 beds	118 (27.1)

ICU - intensive care unit.

* Respondents were allowed to mention more than one intensive care unit type.

### Delirium assessment and management

When asked about the frequency of delirium assessment in the ICU, 267 (61.2%) respondents reported no assessment at all. Regarding the 169 remaining ICU professionals, the most reported frequencies were once (68, 40.2%) and twice per day (41, 24.3%). The overall use of a validated delirium diagnostic tool for adult ICU patients was 72.8%. When evaluated by profession, these data were reported by 80.9% of nurses compared to 74.3% of physicians (p = 0.59). The Confusion Assessment Method for ICU (CAM-ICU) was the most reported validated diagnostic tool (66.9%), followed by the Intensive Care Delirium Screening Checklist (ICDSC) (8.9%). Twenty-one of the 169 respondents (12.4%) reported clinical judgment as the sole delirium assessment method.

Because Brazilian participants constituted more than half of the respondents (n = 270, 61.9%), the delirium assessment was also analyzed by subgroups (Brazil versus other countries), aiming to observe whether Brazilian professionals attitudes differ from those related in other countries. CAM-ICU was the most frequently used diagnostic tool reported by both subgroups but had significantly higher rates with Brazilian respondents (83.0% versus 43.5% for Brazil versus other countries respectively, p < 0.001). Brazil, compared to the other countries, also reported higher overall employment of a validated delirium assessment tool for the ICU (85.0% versus 55.1%, p < 0.001), as well as by its physicians (88.7 x 59.6%, p = 0.001) and physiotherapists (80.8 versus 33.3%, p = 0.010), with no difference regarding nurses when analyzed by profession compared to the other countries. Details concerning delirium assessment are described in table 2.

**Table 2:** Attitudes toward delirium assessment

	All	Brazil	Others	
	(N = 436)	(N = 270)	(N = 166)	p value
	N (%)	N (%)	N (%)	
Delirium evaluation tool*	169 (38.8)	100 (37.0)	69 (41.6)	0.363
Clinical judgment	53 (31.4)	27 (27.0)	26 (37.7)	0.177
CAM-ICU	113 (66.9)	83 (83.0)	30 (43.5)	< 0.001
DRS	13 (7.7)	3 (3.0)	10 (14.5)	0.008
ICDSC	15 (8.9)	4 (4.0)	11 (15.9)	0.011
MMSE	2 (1.2)	0	2 (2.9)	0.165
Proportion of delirium assessment using a validated diagnostic tool for the ICU^a^	123 (72.8)	85 (85.0)	38 (55.1)	< 0.001
Physician	78/105 (74.3)	47/53 (88.7)	31/52 (59.6)	0.001
Physiotherapist	24/37 (64.9)	20/25 (80.8)	4/12 (33.3)	0.010
Nurse	17/21 (80.9)	15/18 (83.3)	2/3 (66.7)	0.489
Other	4/6 (66.7)	3/4 (75)	1/2 (50)	1.000
Number of times delirium is assessed (per day)^b^				
0	4 (2.4)	0 (0)	4 (5.8)	0.026
1	68 (40.2)	42 (42.0)	26 (37.7)	0.633
2	41 (24.3)	25 (25.0)	16 (23.2)	0.856
3	35 (20.7)	24 (24.0)	11 (15.9)	0.248
> 3	14 (8.3)	8 (8.0)	6 (8.7)	1.000

CAM-ICU - Confusion Assessment Method for the Intensive Care Unit; DRS - Delirium Rating Scale; ICDSC - Intensive Care Delirium Screening Checklist; MMSE - Mini Mental State Examination; ICU - Intensive Care Unit.

* Frequencies for each delirium evaluation method described above refer either to its use in isolation or in combination with another tool.

a "Validated diagnostic tool for the ICU" refers to CAM-ICU and ICDSC according to the "Clinical Practice Guidelines for the Management of Pain, Agitation, and Delirium in Adult Patients in the Intensive Care Unit" published in 2013.^(18)^

b N Sum (%) does not equal 169 (100) because 7 participants did not answer this question.

**Figure 1:**
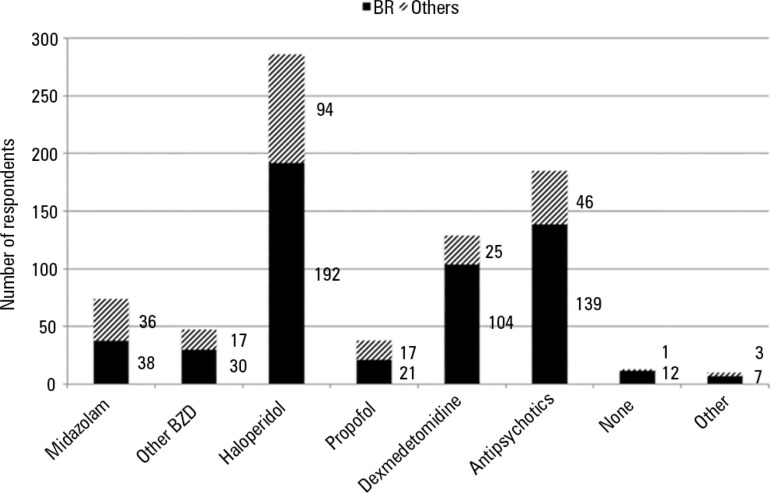
Medications reported by respondents as treatment for delirium symptoms. BZD - benzodiazepines.

Regarding the treatment of delirium symptoms, the most frequent drugs chosen were haloperidol (65.5% of respondents) and antipsychotics (42.4%), followed by dexmedetomidine (29.6%) and midazolam (17%). Chlorpromazine, clonidine, fentanyl, quetiapine, ketamine and non-pharmacological measures were cited by participants as "other." Haloperidol and antipsychotics remained the most frequent drugs that were reported by both subgroups when an analysis by country was performed. The third most frequently reported drug by Brazilian respondents, however, was dexmedetomidine (38.5% versus 15.1%, p < 0.001); participants from other countries reported a higher use of midazolam (21.7% versus 14.1%, p = 0.049). Data are depicted in figure 1.

### Attitudes associated with delirium in subjects requiring noninvasive ventilation

**Figure 2:**
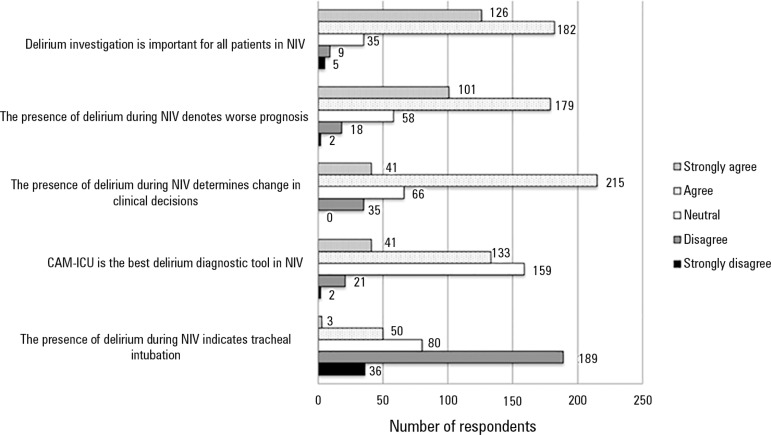
Respondents' perceptions regarding delirium in patients under noninvasive ventilation. NIV - noninvasive ventilation; CAM-ICU - Confusion Assessment Method for the Intensive Care Unit.

To study the influence of delirium on attitudes toward patients requiring NIV, survey participants were asked if they allow NIV in patients with previous delirium diagnosis, if a routine assessment for delirium is performed during NIV and which diagnostic tool is chosen for those patients. Respondents were also asked about their attitudes and perceptions toward potential adverse outcomes in case of delirium onset during NIV (Figure 2).

Concerning the indication of NIV in patients already presenting with delirium, 16.3% of all (436) respondents never allow the use of NIV in this clinical context, while 44.5% answered "sometimes". Almost 42% of respondents agree and 28.9% strongly agree that delirium assessment should be performed in all patients during NIV; however, this practice is routinely applied by only 31.5% of participants. Thirty-six percent of participants remained "neutral" when asked if CAM-ICU is the best delirium diagnostic tool in patients during NIV, while only 9.4% strongly agree.

With respect to patients who are submitted to NIV without a previous diagnosis of delirium, nearly 55% (n = 239) of respondents proceed to delirium evaluation in the onset of agitation. Clinical judgment is the diagnostic tool chosen by 46% of these 239 respondents, and CAM-ICU is chosen by 41.8%. If delirium is diagnosed, 56.8% of respondents use haloperidol as treatment, followed by dexmedetomidine (used by 35.6%). Midazolam was cited as the treatment used by 14.2% of respondents and other benzodiazepines by 8.4%.

NIV failure, however, motivates 40.1% of all respondents to perform a delirium assessment. It is believed by 64.1% of participants that the presence of delirium during NIV denotes a worse prognosis, and 58.7% of respondents agree that it determines a change in clinical decisions. When asked about interventions, if delirium is diagnosed during NIV, 63.3% of respondents claim to use pharmacological intervention, while 31.9% choose to interrupt NIV and 16.7% proceed to tracheal intubation.

Similar perceptions and attitudes can be observed between Brazilian respondents and those from other countries with respect to delirium in patients during NIV. Disagreement could be noticed only in two circumstances: when professionals were asked whether they agree that the presence of delirium determines changes in clinical decisions (Brazilian respondents disagree in 10.7% versus 3.6%, p = 0.010) and if delirium during NIV indicates tracheal intubation (Brazilian respondents strongly disagree in 11.1% versus 3.6%, p = 0.007).

## DISCUSSION

We conducted a multinational survey aiming to characterize the attitudes of ICU professionals, mostly from South America and Europe, toward delirium assessment and management, as well as clinical decisions regarding delirious patients requiring NIV.

Despite increased knowledge that delirium is common and related to poor outcomes in ICU patients, this condition is still underdiagnosed by healthcare professionals, and delirium monitoring using a validated diagnostic tool is markedly neglected as shown in previous surveys.^(1,8)^ It is also known that standard clinical evaluations do not allow for an accurate diagnosis of delirium. When based solely on clinical perception, non-psychiatrist physicians may underdiagnose up to 3/4 of all ICU delirium cases, particularly in its hypoactive forms.^(16)^ Systematic monitoring is therefore necessary for the identification of risk factors and clinical manifestations of this condition.^(17)^ The recent Society of Critical Care Medicine/Pain, Agitation and Delirium (SCCM/PAD) guidelines recommend routine monitoring of delirium in adult ICU patients, at least once per nursing shift.^(18)^ For this purpose, CAM-ICU and ICDSC are the most valid and reliable diagnostic tools because both instruments show high sensitivity and specificity when compared to gold-standard criteria (Diagnostic and Statistical Manual of Mental Disorders - DSM- IV according to the American Psychiatric Association), high inter-rater reliability and clinical feasibility.^(19-21)^ Both instruments can be applied in non-verbalizing patients and were translated into and validated in several languages, including Portuguese,^(22)^ allowing wide employment in clinical practice.

In the present survey, 38.8% (n = 169) of respondents reported systematic delirium assessment in their ICU; an assessment frequency of at least twice per day was reported by 53.3% (n = 90) of those participants. This result demonstrates a substantial gap in knowledge translation into practice. Clinical evaluation, alone or combined with another method, was considered a diagnostic tool by 31.4%. CAM-ICU was the most frequent validated instrument reported (66.9%), while ICDSC was mentioned by 8.9%.

Our results differ in some aspects when compared to previous surveys. We found lower rates of delirium assessment, but from those who reported systematic evaluation, higher rates were mentioned considering the employment of a specific delirium diagnostic tool, as well as a higher frequency of delirium monitoring per day.

Patel et al. conducted a follow-up study including 1384 healthcare professionals from North America,^(8)^ aiming to assess current behaviors and attitudes regarding delirium and sedation practices and to identify changes in behaviors and attitudes regarding delirium since the original survey, conducted five years earlier by the same group.^(23)^ In the follow-up study, the authors found higher rates of delirium assessment (59% versus 49%) and nearly a three-fold higher rate regarding use of a specific screening tool (33% versus 12%, p < 0.001). Salluh et al. conducted a cross-sectional survey, aiming to characterize the practices of Brazilian ICU physicians toward sedation and delirium. From a total of 1015 respondents, 91.3% relied on the clinical evaluation for delirium assessment. An evaluation frequency of at least twice per day was reported by 34.7%.^(1)^ In our survey, the most frequently reported diagnostic tool was CAM-ICU, while only 27 out of 100 Brazilian respondents reported the employment of clinical evaluation for delirium assessment. Delirium evaluation frequency of at least twice a day was reported by 57% by this subgroup.

Finally, a prospective, observational, multicenter, multinational study was recently published as a two-part survey that included data from 101 hospitals (part 1) and 868 patients (part 2). An implementation rate of delirium assessment with a validated score was initially described as 44%. Analysis from part 2, however, revealed that in actual practice, only 27% of included patients were actually monitored with a validated score.^(24)^

Concerning the treatment of delirium symptoms, there is some variability between studies. Haloperidol was the most frequently reported drug in our study (65.5% of respondents), followed by atypical antipsychotics (42.4%) and dexmedetomidine (29.6%). Interestingly, the use of benzodiazepines was mentioned by 24.8% of participants (n = 108). The study by Patel et al. found haloperidol to be the most reported drug (86%), followed by antipsychotics (40%). Benzodiazepines, however, were used at a higher rate (near 40%),^(8)^ which was also observed in the Brazilian survey conducted by Salluh et al.,^(1)^ where benzodiazepines were considered to be the treatment option of 42.3% of physicians. In contrast, Luetz et al. observed that antipsychotics were the most frequently used agents (99%), and 82% of ICUs used benzodiazepines as part of their treatment regime.^(24)^

According to current SCCM/PAD guidelines,^(18)^ evidence that haloperidol or other antipsychotics are associated with improved outcomes remains to be unequivocally established. Considering the association of benzodiazepines with delirium onset in ICU patients, guidelines suggest continuous intravenous infusions of dexmedetomidine as the sedation strategy rather than benzodiazepine infusions to reduce the duration of delirium in adult ICU patients with delirium unrelated to alcohol or benzodiazepine withdrawal.^(25,26)^

In regards to attitudes associated with delirium in subjects requiring NIV, our study demonstrated a significant discrepancy between recognizing the importance of delirium assessment during NIV and its actual clinical application. While almost 71% of respondents agree that delirium evaluation should be performed in all ICU patients, this practice is routinely applied by only 31.5% of all participants. Moreover, when asked if CAM-ICU is the best delirium diagnostic tool in patients during NIV, only 9.4% strongly agree.

Finally, NIV failure motivates less than half (40.1%) of the respondents to perform a delirium assessment, although 64.1% believe that the presence of delirium during NIV denotes a worse prognosis. Our data may encourage professionals to better investigate the possible influence of delirium in NIV failure in the ICU setting. It is known that, for the last two decades, an increasing number of studies aimed to provide safe indications of NIV in patients presenting with acute renal failure that was precipitated by causes other than chronic obstructive pulmonary disease exacerbation, the first consistent evidence of NIV benefits over MV.^(27)^ In contrast, it was already demonstrated that NIV failure (and delay for its identification) is associated with increased morbi-mortality.^(11,28)^ These findings motivated several studies to identify potential predictors of NIV failure,^(28,29)^ but delirium was seldom evaluated. Nevertheless, it can be assumed that patient cooperation and tolerance, as well as preserved mental status, are attributes reported to be necessary for effective ventilation,^(29)^ and these characteristics may be compromised in delirious patients. In 2012, Charlesworth et al. performed a systematic review and meta-analysis of the literature to determine the prevalence of delirium in patients receiving NIV for acute renal failure and to quantify the prognostic impact of delirium with respect to NIV. A literature search retrieved only three articles, reflecting poor research in this area. Despite the absence of high-quality studies, results from a meta-analysis should encourage more studies regarding delirious patients requiring NIV, as the pooled risk ratio for NIV failure was found to be 2.12 (95% CI 1.41-3.18).^(30)^

Our survey has some limitations that should be mentioned. Although multinational and multicenter, questionnaires analyzed retrieved information mainly from professionals who resided in Brazil, despite wide electronic distribution of questionnaires by a mailing list, aiming to reach a higher number of countries in Latin America and Europe.

Perceived attitudes and perceptions described cannot, therefore, be adequately generalized. Nonetheless, comparisons between Brazilian professionals and those from other countries were performed, aiming to describe potential differences in the perceptions and attitudes of ICU professionals.

The other countries from which questionnaires were answered comprised not only different geographic areas but also potentially different organizational and financial aspects of ICU management that could interfere with professional care. It is known, however, that delirium diagnosis and management can be achieved without higher costs with the application of simple and non-expensive diagnostic tools mentioned in our study (CAM-ICU and ICDSC) that are easily reproducible by different categories of ICU professionals; these tools have also been translated into different languages and are validated by current SCCM/PAD guidelines.^(18)^ For those reasons, delirium diagnosis (and subsequent management) is feasible in a wide range of institutions, despite their financial and geographic aspects, minimizing any interference on our study objectives and results.

Some questionnaires were not answered completely (n = 82). Although the questionnaire was built so that each core question had to be completed before the next was answered, some respondents did not complete the questionnaire regarding secondary issues on the topics.

Analysis of previously published surveys in this field demonstrates similar aspects regarding the total number of participants, a variable description of the number of invitations sent, and the number of surveys completed, as follows: Patel et al.,^(8)^ aiming to assess ICU professionals' behaviors and attitudes regarding delirium and sedation practices and to identify changes in behaviors and attitudes since 2001 (when a similar survey was performed by the same study group), chose to distribute the survey to a convenience sample. Neither the overall number of questionnaires distributed nor the number of incomplete forms were mentioned. Devlin et al.,^(31)^ aiming to evaluate the attitudes and perceptions of intensive care nurses with respect to delirium assessment, described how surveys were distributed through hospitals; the total number of questionnaires sent (601) and the response rate (55%) were also cited. Finally, Luetz et al.^(24)^ conducted a two-part survey (the first part contained general information from participating ICUs, followed by a second part referring to patient data), of which the primary aim was to investigate the implementation rate of delirium monitoring among intensivists. Authors reported that out of the potential 567 questionnaires for the first part, 528 were not submitted. From the 129 submitted questionnaires, 28 were incomplete - a 21.7% loss. With respect to the second part, 1004 questionnaires were distributed, from which 868 were included in the analysis - a 13.5% loss.

Finally, because the study design is characterized by closed questions, discrepancies encountered between the perception that delirium recognition is important and the low rates of delirium assessment cannot be better explored in the study. The gap between the perceived importance of delirium evaluation and its practice, although described in previous surveys as mentioned in the discussion, can only become evident after questionnaire analysis; the option for respondents to justify their answers and disagreements regarding delirium assessment performance and choices regarding diagnostic tool was not available.

## CONCLUSION

This survey provides data that strongly reemphasizes poor efforts toward delirium assessment and management in the intensive care unit setting, especially regarding patients under noninvasive ventilation. Regarding the scarce data from the literature with respect to delirium impact on noninvasive ventilation failure, our study provides valuable information about perceived attitudes of intensive care unit healthcare professionals in this field. The results presented should therefore encourage educational efforts for the implementation of evidence-based strategies for the management of critically ill patients who might potentially be at a higher risk of noninvasive ventilation failure if delirium symptoms are accurately identified.
