# Supplementary material for: Delirium in intensive care unit patients under noninvasive ventilation: a multinational survey
Source: Rev Bras Ter Intensiva. 2015 Oct-Dec;27(4):360–8. doi: 10.5935/0103-507X.20150061 (PMC4738822; doi:10.5935/0103-507X.20150061)
Supplement: Supplementary file 1 [file rbti-27-04-0360-suppl01.pdf]

Delirium in intensive care unit patients under noninvasive ventilation: a multinational survey

Delirium em pacientes na unidade de terapia intensiva submetidos à ventilação não invasiva: um inquérito multinacional

Lilian Maria Sobreira Tanaka[1], Jorge Ibrain Figueira Salluh[2], Felipe Dal-Pizzol[3], Bruna Brandão Barreto[4], Ricardo Zantieff[5], Eduardo Tobar[6], Antonio Esquinas[7], Lucas de Castro Quarantini[4,8], Dimitri Gusmao-Flores[4,5]

Appendix 1 - Survey

Delirium in patients under noninvasive ventilation

To the esteemed critical care specialist/professional,

You are invited to answer this quick survey that takes less than minutes to complete. These data will be fundamental to understand the routine of managing delirium in patients under noninvasive ventilation worldwide, which will positively impact the care given to these patients.

In advance, we offer our sincere appreciation.

International Delirium Research Consortium

Mark with an "X" all that apply.

1. Occupation
( ) Physician
( ) Nurse
( ) Physiotherapist

2. Specialist in critical care:
( ) Yes
( ) No

3. Years of practice in critical care:
( ) 1 - 5 years
( ) 5 - 10 years
( ) > 10 years

4. Main place of work:
( ) Public Hospital
( ) University Hospital/Teaching Hospital
( ) Private Hospital

Continue...

... continuation

5. Types of intensive care units (ICU)
( ) Medical
( ) Cardiac
( ) Surgical
( ) Mixed
( ) Neuro
( ) Trauma
( ) Other:____

6. Number of beds in your ICU:
( ) up to 10
( ) 11 - 20
( ) > 20

7. What is the average occupancy rate of your ICU (based on previous year)?
( ) < 50%
( ) 50 - 75%
( ) 75 - 85%
( ) > 85%

8. What is the approximate daily percentage of mechanically ventilated patients in your ICU (based on previous year)?
( ) < 20%
( ) 20 - 40%
( ) 40 - 70%
( ) > 70%

9. Do you or your ICU assess the presence of delirium in a systematic way, at least once per day, with a validated diagnostic tool?
( ) Yes
( ) No

Continue...

... continuation

10. If yes, which instrument do you use (check all that apply)?
( ) General clinical evaluation
( ) CAM-ICU
( ) Delirium rating scale
( ) Intensive Care Delirium Screening Checklist
( ) MMSE
( ) Other: _______________

11. How many times is the presence of delirium assessed in your ICU each day?
( ) 0
( ) 1
( ) 2
( ) 3
( ) > 3

12. Which drug (s) do you normally use to treat delirium (check all that apply)?
( ) midazolam
( ) other benzodiazepine
( ) haloperidol
( ) propofol
( ) dexmedetomidine
( ) atypical anti-psychotic (olanzapine, quetiapine, clozapine, risperidone)
( ) other: _________________.

13. The assessment of delirium is routinely performed during the procedure of noninvasive ventilation:
( ) Yes
( ) No

14. If yes, what is the diagnostic tool used?
( ) General clinical evaluation
( ) CAM-ICU
( ) Delirium rating scale
( ) Intensive Care Delirium Screening Checklist
( ) MMSE
( ) Other: _______________

15. If the patient presents agitation during noninvasive ventilation (NIV), is a delirium assessment made?
( ) Yes
( ) No

Continue...

... continuation

16. If yes, what is the diagnostic tool used?
( ) General clinical evaluation
( ) CAM-ICU
( ) Delirium rating scale
( ) Intensive Care Delirium Screening Checklist
( ) MMSE
( ) Other: _______________

17. If the patient presents interaction failure during noninvasive ventilation (NIV), is a delirium assessment made?
( ) Yes
( ) No

18. If yes, what is the diagnostic tool used?
( ) General clinical evaluation
( ) CAM-ICU
( ) Delirium rating scale
( ) Intensive Care Delirium Screening Checklist
( ) MMSE
( ) Other: _______________

19. If, after adjusting the NIV the patient presents agitation or interaction failure, what drug do you usually use (check all that apply)?
( ) midazolam
( ) other benzodiazepine
( ) haloperidol
( ) morphine
( ) fentanyl
( ) propofol
( ) dexmedetomidine
( ) atypical anti-psychotic (olanzapine, quetiapine, clozapine, risperidone)
( ) none
( ) other: _______________________.

20. If the patient presents delirium during noninvasive ventilation, you (check all that apply):
( ) use pharmacological treatment
( ) stop the proceedure
( ) performs intubation.
( ) other: _______________

Continue...

... continuation

21. If the diagnosis of delirium is given during noninvasive ventilation, what drug do you usually use to treat it (check all that apply)?

( ) midazolam

( ) other benzodiazepine

( ) haloperidol

( ) morphine

( ) fentanyl

( ) propofol

( ) dexmedetomidine

( ) atypical anti-psychotic (olanzapine, quetiapine, clozapine, risperidone)

( ) none

( ) other: ______________________.

22. In patients diagnosed with delirium, you allow noninvasive ventilation to be used:

( ) Never

( ) Always

( ) Sometimes. In which situation?______________________.

23. Regarding the following statements about delirium in patients on NIV, answer:

It is important to investigate delirium in all patients on NIV

( ) Strongly disagree ( ) Disagree ( ) Neither
( ) Agree ( ) Totally agree

The presence of delirium during NIV represents worse prognosis

( ) Strongly disagree ( ) Disagree ( ) Neither
( ) Agree ( ) Totally agree

The presence of delirium during NIV determines behavior change

( ) Strongly disagree ( ) Disagree ( ) Neither
( ) Agree ( ) Totally agree

The CAM-ICU is the best tool to diagnose delirium in patients on NIV

( ) Strongly disagree ( ) Disagree ( ) Neither
( ) Agree ( ) Totally agree

The presence of delirium during NIV indicates the need for endotracheal intubation

( ) Strongly disagree ( ) Disagree ( ) Neither
( ) Agree ( ) Totally agree

Appendix 2S - List of all participating countries and the respective number of respondents.

Country	Number of respondents (%)
Afghanistan	1 (0.2)
Armenia	1 (0.2)
Austria	2 (0.5)
Australia	2 (0.5)
Belgium	2 (0.5)
Bulgaria	1 (0.2)
Brazil	270 (61.9)
Canada	1 (0.2)
Chile	16 (3.7)
Colombia	6 (1.4)
Costa Rica	1 (0.2)
Germany	1 (0.2)
Denmark	2 (0.5)
Ecuador	1 (0.2)
Egypt	1 (0.2)
Spain	9 (2.1)
France	4 (0.9)
Greece	1 (0.2)
Hong Kong	2 (0.5)
India	2 (0.5)
Italy	21 (4.8)
Japan	2 (0.5)
Mexico	2 (0.5)
Panama	1 (0.2)
Peru	5 (1.1)
Philippines	3 (0.7)
Poland	1 (0.2)
Portugal	15 (3.4)
Russian Federation	1 (0.2)
Saudi Arabia	1 (0.2)
Sudan	1 (0.2)
Singapore	1 (0.2)
Tunisia	2 (0.5)
Turkey	38 (8.7)
United Kingdom	2 (0.5)
United States	12 (2.8)
Uruguay	1 (0.2)
Yugoslavia	1 (0.2)
